# Extended Distal Pancreatectomy for Cancer of the Body and Tail of the Pancreas: Analysis of Early and Late Results

**DOI:** 10.3390/jcm12185858

**Published:** 2023-09-08

**Authors:** Cosimo Sperti, Simone Serafini, Alberto Friziero, Matteo Todisco, Giulia Tamponi, Domenico Bassi, Amanda Belluzzi

**Affiliations:** 1 Hepatobiliary Surgery and Liver Transplantation Unit, Department of Surgery, Oncology and Gastroenterology, 2nd Surgical Clinic, University of Padua, 35122 Padova, Italy; simone.serafini@aopd.veneto.it (S.S.); domenico.bassi@aopd.veneto.it (D.B.); 2 1st Surgical Clinic, Department of Surgery, Oncology, Gastroenterology, University of Padua, 35122 Padova, Italy; alberto.friziero@aopd.veneto.it (A.F.); giulia.tamponi@aopd.veneto.it (G.T.); amanda.belluzzi@aopd.veneto.it (A.B.); 3 Radiology Unit, Department of Radiology, University of Padua, 35128 Padova, Italy; matteo.todisco@aopd.veneto.it

**Keywords:** pancreas, pancreatic neoplasms, distal pancreatectomy, extended pancreatectomy, complications, follow-up

## Abstract

Cancer of the body-tail of the pancreas often involves adjacent structures. Thus, surgical treatment may be extended to other organs or vessels in order to achieve radical resection. The aim of this study is to evaluate the safety and efficacy of extended distal pancreatectomy for ductal adenocarcinoma of the body and tail of the pancreas. Between January 2000 and December 2016, 101 patients underwent distal pancreatectomy (DP) for pancreatic cancer: 65 patients underwent standard-DP and 36 extended-DP, including the resection of the partial stomach (*n* = 12), adrenal gland (*n* = 7), liver (*n* = 7), colon (*n* = 8), celiac axis (*n* = 6), portal vein (*n* = 5), jejunum (*n* = 4) and kidney (*n* = 4). The two groups were compared in terms of their TNM classification, pathological grade, nodal status, state of resection margins, age, sex and levels of preoperative serum carbohydrate antigen 19-9 (CA 19.9). The morbidity and mortality were not statistically different in the two groups. The two groups disease-free and overall survival rates were significantly influenced by the tumor’s stage, nodal status, pathological features and resection margins. Survival was not influenced by the extent of the surgical resection. However, when patients were stratified according to the type of extended resection, survival was worse in the group of patients undergoing vascular resection. Multivariate analysis showed that the stage and resection margins are independent predictors of disease-free and overall survival. Extended distal pancreatectomy may be performed with acceptable morbidity and mortality rates. Survival is not significantly different after standard or extended resection. However, the rate of tumor recurrence is high, and long-term survival is a rare event, especially in those patients who undergo distal pancreatectomy associated with vascular resection.

## 1. Introduction

Traditionally, pancreatic cancer arising in the distal pancreas has been regarded to be more aggressive compared to the proximal part of the pancreas [1,2]; the delayed symptoms can explain the detection of the tumor at more advanced stages [3]. Distal pancreatectomy (DP) with splenectomy is the surgical procedure of choice for malignant lesions arising in the body and tail of the pancreas. In recent years, the 30-day operative mortality after DP has declined by <5% in high-volume centers [4,5], while the operative morbidity remains significant at up to 50% [6,7]. The surgical management of body-tail pancreatic cancer is sometimes challenging due to the late onset of unspecific symptoms, resulting in the frequent presence of a higher burden of disease. Therefore, multi-visceral resection is often required to achieve radical resection.

The body-tail pancreatic cancer spreads most frequently tothe stomach, colon, kidney, adrenal gland, liver and the adjacent vascular structures [8,9]. However, little data are available regarding the current indications and early and late outcomes following the extended resection of distal pancreas. The purpose of this study is to review a single institution’s experience with standard and extended DP for pancreatic cancer and the short- and long-term outcomes associated with these procedures in order to define the current indications for surgical treatment. The rate and sites of tumor recurrence after operation were also analyzed.

## 2. Materials and Methods

Between January 2000 and December 2016, 101 patients who underwent DP for adenocarcinoma of the pancreas in a single institution were identified from a prospective maintained surgical database. All patients’ data were collected retrospectively from the patients’ clinical notes, operative records, pathologic results and follow-up. Patients with cystic neoplasms, IPMNs, islet cell tumors or pancreatic involvement by extra-pancreatic neoplasms were excluded from the study. CT with angiography reconstruction was the imaging of choice for the tumor staging. All patients underwent standard lymph node dissection including 8a, 9, 10, 11, 18 nodal stations [10]. Resections were defined as curative (R) when the pathology report confirmed negative resection margins or R1 in the presence of tumors <1 mm from the resection margins, according to the Leeds criteria [11]. Tumors were staged according to the Union for International Cancer Control (UICC) TNM classification [12]. Each patient’s clinical and pathological records were reviewed, and the following characteristics were included in our analysis: age, sex, type of surgery (standard or extended distal pancreatectomy), preoperative serum CA 19-9 levels (RIA, Centocor Inc., Malvern, PA, USA, reference: <37 kU/L), tumor stage, lymph node status, pathological grade, RO resection, type of recurrence, disease-free survival and overall survival. Disease-free survival (DFS) was measured from the date of surgery to the date of radiologically detected recurrence or censoring. Overall survival (OS) was measured from the date of surgery to the date of death or censoring. All patients underwent regular follow-up, which included a physical examination, abdominal CT or US and tumor marker assay every 3 months for the first 2 years, and every 6 months thereafter. Postoperative complications were recorded according to the guidelines proposed by Dindo et al. [13] and De Oliveira et al. (Clavien-Dindo classification) [14]. Delayed gastric emptying or postoperative ileus was defined as the inability to tolerate a regular diet or the need for a nasogastric tube more than 10 days postoperatively. Mortality was defined as death within the same hospitalization or within 30 days of discharge. Pancreatic fistula was defined and graded according to the system proposed by the International Study Group of Pancreatic Fistula (ISGPF) [15]. Any drain output with an amylase content more than 3 times the upper limit of the normal serum amylase fluid level at postoperative day 3 or later was considered a pancreatic fistula (PF). The extent of surgery was classified as DP with splenectomy or multi organ resection DP. A multiorgan resection was defined as any DP in which any other intra-abdominal organ was resected concomitantly (except for cholecystectomy) or vascular resection, according to the International Study Group of Pancreatic Surgery (ISGPS) [8]. Although liver involvement is considered distant metastasis, liver resection is included in the group of extended resections [8]. Only young patients with good health condition and isolated liver metastasis requiring wedge resection were included in our study. When arterial (celiac axis) resection was performed, the proper hepatic artery was palpated and controlled through intraoperative ultrasound Doppler after celiac artery occlusion. The celiac artery and the common hepatic artery were then transected; the right gastroepiploic and the right gastric artery were preserved together with the whole stomach. We did not perform preoperative embolization of hepatic artery or left gastric artery. The method of pancreatic stump closure was based on the surgeon’s preference, and this included hand-sutured closure, linear stapled closure and linear stapled closure with buttressing. Whenever possible, the main pancreatic duct was identified and stitched. Every patient received at least one intrabdominal drain to measure the postoperative amylase levels and assess its output in the postoperative course. Postoperative somatostatin analogues were administered selectively. Patients were monitored in the immediate postoperative period in the specialized surgical Intensive Care Unit (ICU). If pancreatic fistula occurred, the patient was maintained on a regular oral diet. Clinically significant PF (grade B or C) was managed with antibiotics and US-guided percutaneous drainage until PF resolution. Parenteral nutrition or somatostatin analogues were used infrequently. Adjuvant therapy included gemcitabine-based chemotherapy or, in recent years, combination chemotherapy (FOLFIRINOX or Gemcitabine + Nab-paclitaxel). Radiotherapy was administered in selected cases. Neoadjuvant therapy was provided more recently in some locally advanced tumors.

Statistical analyses were run using STATA, version 14.1 (College Station, TX, USA). Receiver operating characteristic (ROC) curve analysis was used to ascertain the optimal cut-off for predicting DFS and OS after pancreatectomy. The optimal cut-off was identified as the point of intersection nearest the top left-hand corner between the ROC curve and the diagonal line from the top right-hand corner to the bottom left-hand corner of the graph. For the univariate analysis, the patients were divided into two groups: those who underwent standard DP and those who underwent extended DP. Differences between the characteristics of the patients in the two groups were tested for significance using the Mann-Whitney U test, chi-square test, Fisher’s exact test or *t*-student test. All results are presented as median (range). Statistical analyses were performed with SPSS for Windows version 10.0. Univariate and multivariate analysis were used to investigate the effect of the following variables on survival: age, sex, tumor stage, serum CA 19-9 levels, extension of resection, pathological grade, lymph node involvement, resection margins, adjuvant therapy, neoadjuvant therapy. Survival data were estimated using the Kaplan-Meier method and examined using the log-rank test. Multivariate analysis of survival was performed using Cox’s proportional hazards model. Significance was set at *p* < 0.05.

## 3. Results

The patient population had a median age of 69 years (range: 41–87): 53 were females and 48 were males. Only 6 patients were treated with neoadjuvant chemotherapy. Table 1 shows the clinical and pathological details of the 101 patients.

The level of resection was at or to the left of the superior mesenteric vein in 96% of patients. Extended resection was performed in 36 cases (36%): 1 patient had 4; 3 had 3; 10 had 2 and 22 had 1 concomitant organ resection. These organs included the liver (*n* = 7), adrenal gland (*n* = 7), stomach (*n* = 12), portal vein (*n* = 5), celiac axis (*n* = 6), jejunum (*n* = 4), colon (*n* = 8) and kidney (*n* = 4). When patients were grouped by standard DP versus extended DP (Table 2), the two groups did not show any significant difference in terms of age, sex, tumor grade, nodal status, R0 resection or number of patients administered adjuvant therapy.

As expected, the rate of advanced tumors (stage III–IV) was significantly higher in the extended operation group (*p* < 0.001). The mean operative time was 180 min (range: 120–390); the mean estimated blood loss (EBL) was 500 mL. The pancreatic stump was oversewn in 15 patients (60%), stapled in 43 patients (20%) and both stapled and oversewn in 43 patients (20%). R0 resection was achieved in 59 patients: 17 (47%) and 42 (65%) in the extended and standard resection group, respectively. The postoperative course is reported in Table 3. Two deaths occurred in the hospital with a perioperative mortality rate of 2.1%, one following myocardial infarction and the other for intra-abdominal abscess, in the extended and standard resection group, respectively. The overall postoperative complications rate was 35% (35/101 patients): 39% of patients with extended resection and 32% after standard resection. The most common complication (Table 3) was pancreatic fistula (*n* = 12), followed by intra-abdominal fluid collection (*n* = 10) and peritoneal bleeding (*n* = 5); eight patients required a second surgical procedure for bleeding (*n* = 5), colonic fistula (*n* = 2) and abdominal abscess (*n* = 1). Seventy-two patients (72%) underwent adjuvant chemotherapy, including the six patients who had undergone preoperative chemotherapy. Follow-up was available for all patients and ranged from 6 to 216 months.

### 3.1. Recurrence and Disease-Free Survival

**Figure 1:**
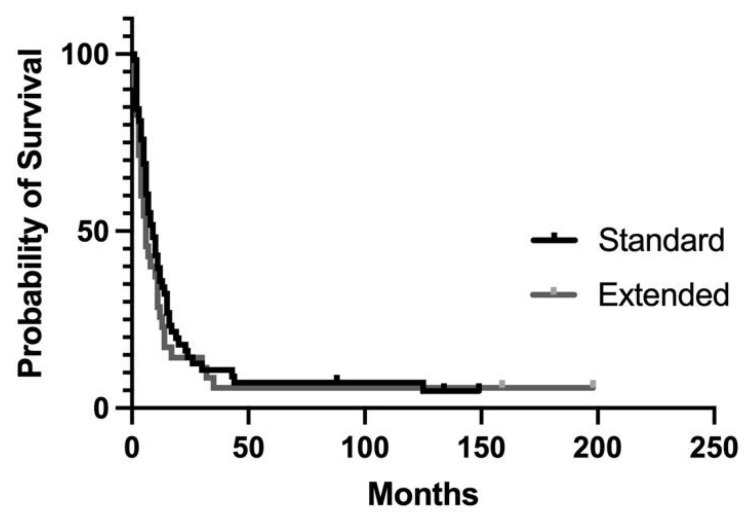
Disease free survival after standard or extended distal pancreatectomy: *p* = 0.309.

With a median follow-up of 6 months (range: 2–44 months), excluding two postoperative deaths, pancreatic cancer recurred in 92/99 patients (92%). The median disease-free survival was 8 months: 6 months after extended DP (range: 2–35 months) and 9 months (range: 2–44 months) after standard DP. The overall disease-free survival was not statistically different in the two groups of patients (Figure 1).

The most frequent site of recurrence was the liver (*n* = 49), followed by the peritoneum (*n* = 36); 18 patients recurred in more than two sites. Twelve patients had other distant recurrences (lung, ovary, bone, para-aortic node). There was no substantial difference in the distribution of the sites of recurrence between the two groups of patients (Table 4).

Three patients with recurrent disease survived more than 5 years after resection, while one patient is still alive 9 years after primary pancreatic resection and 4 years after ovariectomy for ovarian metastasis.

In the univariate analysis, the stage (*p* < 0.001), nodal status (*p* = 0.009), grading (*p* = 0.03) and R0 resection (*p* < 0.001) correlated significantly with DFS. The multivariate Cox regression analysis showed that only the stage (*p* = 0.03) and resection margin (*p* = 0.002) were independent predictors of DFS (Table 5). 

**Figure 2:**
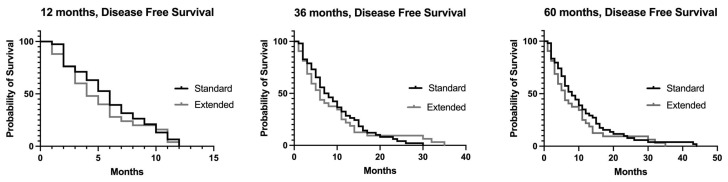
Disease free survival at 12, 36 and 60 months.

When the patients were stratified by the type of resection (visceral or vascular resection), no difference in the DFS was noted. The disease-free survival at 12, 36 and 60 months was not statistically different in the two groups of patients (Figure 2). With the limitation of the small number of patients undergoing arterial or venous resection, there was no difference in the recurrent ratio and disease-free 5-year survival rate between patients who underwent arterial or venous resection (Supplementary Table S2). Loco-regional recurrence occurred in the same proportion in both group of patients (60%).

### 3.2. Overall Survival

**Figure 3:**
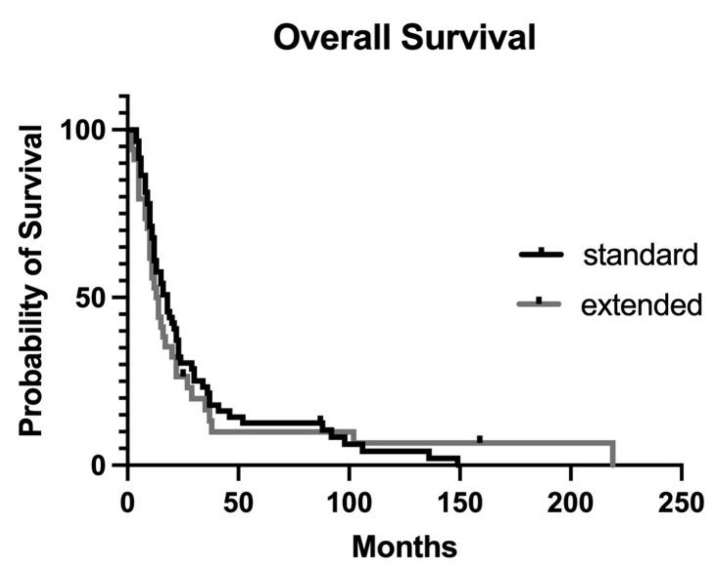
Overall survival after standard or extended distal pancreatectomy: *p* = 0.631.

With a median follow-up of 36 months (range: 6–216 months), excluding two postoperative deaths, 90/99 (91%) patients died of pancreatic cancer, while the other four patients died of unrelated causes. The median OS for the whole cohort was 16 months (range: 3–216 months). As in the case of DFS, extended resection was not an independent predictor of OS (Figure 3).

In the univariate Cox regression analysis, the stage (*p* < 0.001), lymph node status (*p* = 0.03), R0 resection (*p* < 0.001) and vascular resection (*p* = 0.02) were significantly correlated with the OS. The multivariate Cox regression analysis showed that the tumor stage (*p* = 0.01) and margin of resection (*p* = 0.002) were independent predictors for OS (Table 6).

**Figure 4:**
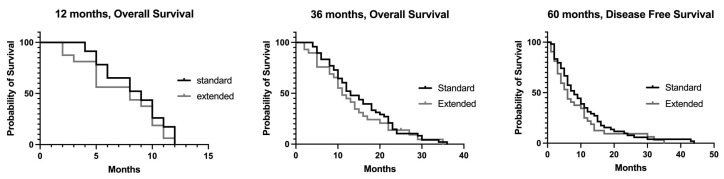
Overall survival at 12, 36 and 60 months.

The survival at 12, 36 and 60 months was not statistically different in the two groups of patients (Figure 4).

When the patients in the extended operation group were stratified according to the type of resection (vascular or visceral resection), the highest survival rate was observed in the DP+ visceral resection group (*p* = 0.02). With the limitation of the small number of patients undergoing arterial or venous resection, there was no difference in the survival rate between the two groups of patients (Supplementary Table S2). If we exclude patients with liver metastases from the analysis, both the disease-free and overall survival were not statistically different between the standard and extended resection groups (Supplementary Figures S1 and S2). At the latest follow-up, five patients were alive (four disease-free): three after standard resection and two after multi-visceral resection. A total of nine patients (9%) survived >5 years after pancreatic resection: five after standard and four after extended (visceral) resection. During the follow-up period, nine patients were re-operated on for recurrent disease. The sites of relapse were: nodal (*n* = 2), nodal and colon (*n* = 1), colon and bladder (*n* = 1), residual pancreas (*n* = 1), liver (*n* = 2), lung (*n* = 1) and ovary (*n* = 1). Radical, palliative and bypass surgery were performed in five, three and one patient, respectively. The median survival after re-operation was 18 months (range: 5–156 months); one patient was still alive 54 months after re-resection.

## 4. Discussion

The ongoing improvement of imaging and diagnostic techniques have resulted in an increase in the preoperative diagnosis of body-tail pancreatic cancer involving adjacent or distant organs [16,17]. In our experience of 101 distal pancreatectomy for adenocarcinoma of the pancreas, 36% of the patients had an associated resection of other structures involved by cancer. Our result is in alignment with the 25–78% range reported by other series [5,18,19], confirming the high tendency to perform more extended and eventually more challenging surgical procedures to achieve R0 resection for cancer located in the distal pancreas [20]. When performing more extended operation for this well-known aggressive cancer, some questions may arise: (1) does the associated resection of other organs or structures increase the risk of operation in terms of mortality and morbidity? (2) Does extended resection modify the rate and type of recurrence when compared to the standard distal pancreatectomy with splenectomy? (3) Is the overall survival impacted by the removal of other organs or vascular structures? The rate of complications (35%) and pancreatic fistula (12%), in our experience, were in line with those previously reported in the literature, and did not show a significant difference in the extended and standard resection groups; two patients died in the postoperative period with a mortality rate of 2.1%. Although the mortality rate after pancreatic resection has decreased considerably in high-volume centers [1], morbidity remains high, as well as after distal pancreatectomy [2,10,11]. The analysis of the literature shows contrasting results. While several single-institution studies showed no increase in mortality with extended resection [19,21,22,23], a recent study reported higher 60-day mortality for patients undergoing extended DP compared to those undergoing a standard DP (4.8% vs. 0.8%), [24]. A study by Siripong et al. [25] looking at morbidity in standard DP versus extended DP reported that the rates of surgical complications were almost 20% greater in extended than standard DP alone. Another study by Burdelski et al. [23] reports a 32% increase in the rate of major complications in extended pancreatectomies versus standard resections. In terms of relative risk, the above-mentioned authors [23] report an almost 6-fold increase in the risk of complications with concurrent colectomy in their univariate analysis, but not in their multivariable analysis. Beetz et al. [26] showed that clinically-relevant pancreatic fistula was more frequent after extended distal pancreatectomy, but without statistical significance. Hartwig et al. [8] found that extended DP was associated with a significant increase in overall and surgical morbidity but not mortality. In a recent report by Tangtawee et al. [27], univariate and multivariate analysis showed that extended resection was not a risk factor for pancreatic fistula or major complications. Thus, it is obvious that morbidity after extended DP may be increased by specific complications related to organ resection, but there are many reports confirming no increase in mortality. Morbidity may potentially affect patients’ survival because patients with major post-operative complications do not receive the recommended adjuvant cancer treatment [28]. Recurrence after curative distal pancreatectomy for pancreatic cancer has rarely been reported separately [29,30]. In our experience, 95% of the patients who underwent resection for distal pancreatic adenocarcinoma presented tumor recurrence, with a median disease-free survival of 8 months. The tumor stage and margin of resection were independent predictors of disease-free survival. In alignment with other reports, our study did not show any significant difference in the recurrence rates between the extended and standard resection groups. Roch et al. reported a 17.4% rate for local recurrence after extended DP versus 11.4% after standard DP (*p* = 0.48); splenic vein thrombosis and invasion were not significantly associated with higher rates of recurrence as local or distant metastases [31].

During the follow-up period, we re-operated on nine patients for recurrent pancreatic cancer, and radical resection was performed on five patients: the median survival after re-operation was 18 months (range: 5–156 months); one patient is still alive 54 months after re-resection. Although the number of patients is low, this result emphasizes the need for the accurate and long-term follow-up of patients who undergo resection for pancreatic cancer and the possible role of surgery in the multimodality management of patients with recurrent cancer. In terms of survival, our study showed that the median survival in patients with extended resection was comparable to the survival after standard resection. However, regarding patients who underwent vascular resection, the overall survival rate was lower. These data are similar to those reported by Beetz et al. [26], who reported lower survival in patients treated with associate vascular resection. Malinka et al. observed a median survival of 29 months in patients with adenocarcinoma who underwent extended DP, which was similar to the survival after standard DP (34 months) [21]. In the work by Hartwig et al., the median survival of 19.8 months after extended DP was comparable to the survival after standard resection [32]. Further support of our results comes from the work of Burdelski et al., which reported a significant survival benefit with extended DP in comparison to palliative surgery (16 vs. 6 months; *p* < 0.005) [23]. In a recent systematic review of the literature, Chandrashekhar et al. [33] analyzed 15 studies focused on extended distal pancreatectomy for pancreatic adenocarcinoma; all but one were retrospective studies. Extended DP was associated with major complications, re-operations and mortality. However, 3- and 5-year survival after extended DP was similar to standard DP. Two recently published articles focused on arterial resection (celiac axis) combined with distal pancreatectomy (DP-CAR). Loos et al. [34] study, from a specialized high-volume German center, focused on 71 consecutive patients, 61% of which received neoadjuvant chemotherapy prior to surgery and 31 patients underwent concomitant venous resection. In total, 32% of the patients developed major complications and 30% of the patients required re-operation, with 30- and 90-day mortalities of 3% and 8%, respectively. The median survival was 28 months, with an encouraging 5-year survival rate of 19.4%. Yoon et al. [35] reported a Korean multicenter study of 75 patients treated with DP-CAR: 56% of the patients underwent neoadjuvant therapy. Twenty (26.7%) patients experienced major complications, with a 4% 90-day mortality. The median disease-free survival was 7 months and the median overall survival was 19 months with a 5-year actuarial OS rate of 24.4% (7 patients surviving at 5-year with an actual survival of 9.3%). The extent of lymphadenectomy during distal pancreatectomy for left-sided pancreatic cancer has been evaluated by Sakamoto et al. [36] in a propensity-score matched multicenter study in Japan. Among 145 patients, 55 patients underwent D1 DP (dissection of 10, 11 and 18 node stations) and 90 underwent D2 DP (dissection of 7, 8, 9, 10, 11, 14 and 18 stations). The long-term survival and the recurrence rate were not significantly different between the two groups, confirming that extended lymphadenectomy does not improve the outcome of patients with left-sided pancreatic cancer.

The main limitations of our study are the retrospective nature of the study and the relatively small sample of patients included. Moreover, the study covers a long period of time, during which adjuvant therapy has been inevitably changed, with new drug combination. A further limitation is the low number of patients who underwent neoadjuvant therapy: therefore, it is reasonable to investigate the role of preoperative therapy in a prospective setting.

## 5. Conclusions

In conclusion, distal pancreatectomy can be performed with acceptable mortality and morbidity rates. Our findings support the hypothesis that extended DP can be safe and effective in the adenocarcinoma of pancreatic body-tail. Nevertheless, in high-volume centers, meticulous patient selection and the proper assessment of the tumor biology and performance status of the patient are paramount to tailor surgical treatment. Finally, a strict postoperative monitoring and follow-up is mandatory to detect possible surgical complications and recurrence, which may impact the mortality and overall survival.

## Figures and Tables

**Table 1:** Demographic and clinical-pathologic characteristics of the patients enrolled in the study.

Variables		
Age, years, median (range)		69 (41–87)
Sex, *n* (%)	Male	48 (48)
	Female	53 (52)
Lymph node, *n* (%)	Negative	34 (34)
	Positive	67 (66)
Grading, *n* (%)	Low Grade	58 (57)
	High Grade	43 (43)
Stage, *n* (%)	I–II	70 (69)
	III–IV	31 (31)
Extended, *n* (%)	No	65 (63)
	Yes	36 (37)
R0 resection, *n* (%)	Yes	59 (58)
	No	42 (42)

**Table 2:** Demographic and clinical-pathologic characteristics compared by type of resection.

Variables		Extended DP	Standard DP	*p*-Value
Age, years, median (range)		69 (41–83)	67.5 (45–87)	0.951
Sex, *n* (%)	Male	16 (45.7)	27 (45)	0.947
	Female	19 (54.3)	33 (55)	
Lymph node, *n* (%)	Negative	12 (34.3)	19 (31.7)	0.795
	Positive	23 (65.7)	41 (67.3)	
Grading, *n* (%)	Low Grade	22 (62.9)	31 (51.7)	0.294
	High Grade	13 (37.1)	29 (48.3)	
Stage, *n* (%)	I–II	13 (37.1)	53 (88.3)	<0.001
	III–IV	22 (62.9)	7 (11.7)	
R0 Resection, *n* (%)	Yes	18 (51.4)	38 (63.3)	0.260
	No	17 (48.6)	22 (36.7)	
CA 19.9, *n* (%)	<37 U/L	25 (69)	40 (62)	0.630
	>37 U/L	10 (31)	20 (38)	
CA 19.9, U/L median (range)		254 (50–1884)	420 (80–1546)	0.096
Morbidity, *n* (%)		12 (33)	19 (29)	0.793
Mortality, *n* (%)		1 (2.9)	1 (1.7)	0.697
Adjuvant CT, *n* (%)		28 (78)	44 (68)	0.464
Neoadjuvant CT, *n* (%)		4 (11)	2 (3)	0.118

DP = Distal pancreatectomy; CT = chemotherapy.

**Table 3:** Complications after resection.

	Extended DP	Standard DP	*p*-Value
POPF, *n*	5 (14)	7 (11)	0.711
Abdominal collection, *n* (%)	5 (14)	5 (8)	0.362
Bleeding, *n* (%)	1 (3)	5 (8)	0.290
Colonic fistula, *n* (%)	1 (3)	1 (1.5)	0.697
Liver abscess, *n* (%)	0	1 (1.5)	0.443
DVT, *n* (%)	0	1 (1.5)	0.443
Pneumonia, *n* (%)	0	1 (1.5)	0.443
DGE, *n* (%)	1 (3)	0	0.194
MI, *n* (%)	1 (3)	0	0.194
Re-operation, *n* (%)	4 (11)	4 (6)	0.420

DP = distal pancreatectomy; POPF: postoperative pancreatic fistula; DVT: deep vein thrombosis; MI myocardial infarction; DGE = delayed gastric emptying.

**Table 4:** Sites of recurrence.

Site	Extended DP	Standard DP	*p*-Value
Liver, *n* (%)	14 (39)	36 (55)	0.060
Local, *n* (%)	10 (28)	12 (18)	0.339
Peritoneum, *n* (%)	12 (33)	13 (20)	0.178
Lung, *n* (%)	3 (8)	4 (6)	0.732
Other, *n* (%)	0	5 (8)	0.079
No recurrence, *n* (%)	4 (11)	4 (6)	0.420

DP = distal pancreatectomy.

**Table 5:** Univariate and multivariate analysis according to disease free survival.

Variables	HR (Univariate)	HR (Multivariate)
Age > 70 years	0.87 (0.57–1.33, *p* = 0.527)	0.77 (0.48–1.22, *p* = 0.261)
Sex	1.08 (0.71–1.64, *p* = 0.722)	0.91 (0.58–1.42, *p* = 0.672)
Lymph node	1.88 (1.17–3.01, *p* = 0.009)	1.51 (0.90–2.52, *p* = 0.118)
Grading	1.60 (1.04–2.45, *p* = 0.031)	1.46 (0.93–2.29, *p* = 0.099)
Stage	2.99 (1.85–4.85, *p* < 0.001)	1.94 (1.04–3.62, *p* = 0.037)
Radicality	3.08 (1.92–4.95, *p* < 0.001)	2.32 (1.35–3.99, *p* = 0.002)
Extended	1.25 (0.81–1.93, *p* = 0.309)	1.14 (0.66–1.98, *p* = 0.630)
Adjuvant Chemotherapy	1.16 (0.75–1.81, *p* = 0.507)	0.72 (0.43–1.20, *p* = 0.205)
Vascular resection	1.64 (0.86–3.13, *p* = 0.132)	0.83 (0.40–1.75, *p* = 0.627)

**Table 6:** Univariate and multivariate analysis according to overall survival.

Variables	HR (Univariate)	HR (Multivariate)
Age > 70 years	0.94 (0.62–1.42, *p* = 0.762)	0.87 (0.56–1.37, *p* = 0.549)
Sex	1.20 (0.79–1.82, *p* = 0.393)	1.05 (0.68–1.63, *p* = 0.814)
Lymph node	1.62 (1.02–2.56, *p* = 0.039)	1.11 (0.67–1.82, *p* = 0.690)
Grading	1.47 (0.96–2.25, *p* = 0.077)	1.37 (0.88–2.13, *p* = 0.166)
Stage	2.96 (1.84–4.76, *p* < 0.001)	2.32 (1.21–4.45, *p* = 0.011)
Radicality	3.50 (2.15–5.71, *p* < 0.001)	2.57 (1.49–4.44, *p* = 0.001)
Extended	1.11 (0.72–1.72, *p* = 0.631)	0.75 (0.43–1.31, *p* = 0.315)
Adjuvant Chemotherapy	1.23 (0.79–1.92, *p* = 0.348)	1.01 (0.63–1.63, *p* = 0.960)
Vascular resection	2.11 (1.09–4.05, *p* = 0.026)	1.32 (0.62–2.80, *p* = 0.469)

## Data Availability

The data presented in this study are available on request from the corresponding author. The data are not publicly available due to ethical restrictions.

## Supplementary Materials

The following supporting information can be downloaded at: https://www.mdpi.com/article/10.3390/jcm12185858/s1, Table S1: Survival analysis of patients who underwent standard resection (Standard group), visceral organ resection (Visceral resection group) and vascular resection (Vascular resection group); Table S2: Survival analysis of patients who underwent arterial resection (Arterial group) and portal vein resection (Venous group); Figure S1: Disease free survival of extended and standard distal pancreatectomy after excluding patients with liver metastases; Figure S2: Overall survival of extended and standard distal pancreatectomy after excluding patients with liver metastases.
