# Supplementary material for: Extended Distal Pancreatectomy for Cancer of the Body and Tail of the Pancreas: Analysis of Early and Late Results
Source: J Clin Med. 2023 Sep 8;12(18):5858. doi: 10.3390/jcm12185858 (PMC10532237; doi:10.3390/jcm12185858)
Supplement: Supplementary file 1 [file jcm-12-05858-s001.zip › jcm-2567623-supplementary.pdf]

Table S1. Survival analysis of patients who underwent standard resection (Standard group), visceral organ resection (Visceral resection group) and vascular resection (Vascular resection group).

Variables	Standard	Extended (visceral resection group)	Extended (vascular resection group)	P value
Disease free survival, months, median (IQR)	9 (2-44)	6 (2-35)	6 (4.5-9)	0.35
Overall Survival, months, median (IQR)	16 (10-132)	14 (3-216)	10 (8-27)	0.02

Table S2: Survival analysis of patients who underwent arterial resection (Arterial group) and portal vein resection (Venous group).

Variables	Arterial Group (n=6)	Venous Group (n=5)	P value
Disease free survival, months, median (IQR)	8 (4.5-12)	6 (5-6)	0.62
Overall Survival, months, median (IQR)	11 (8-27)	12 (9-16)	0.98

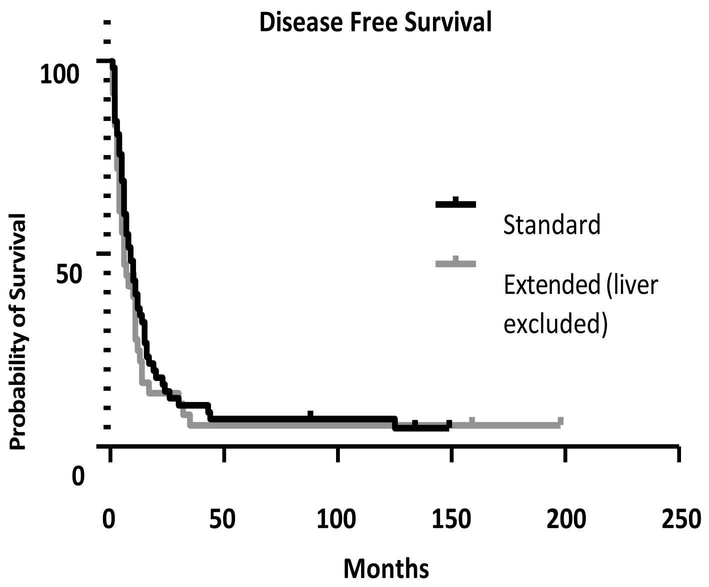

Figure S1. Disease free survival of extended and standard distal pancreatectomy after excluding patients with liver metastases

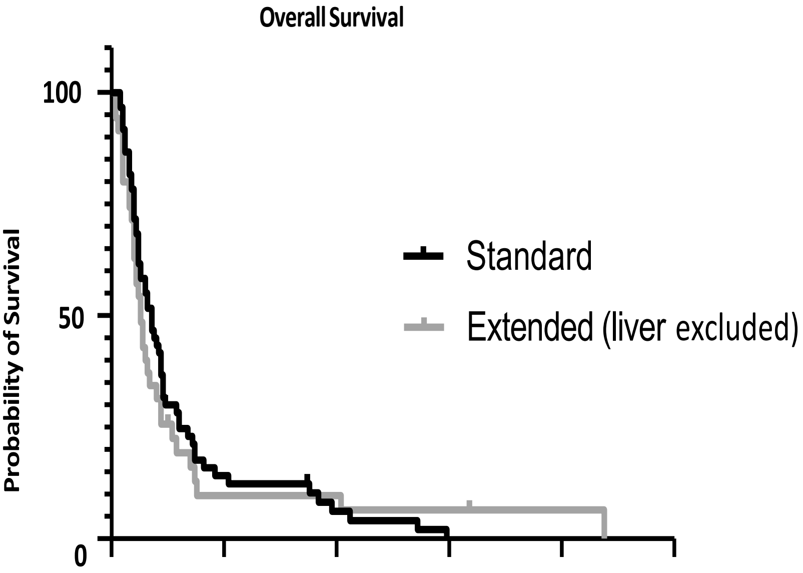

Figure S2. Overall survival of extended and standard distal pancreatectomy after excluding patients with liver metastases
